# Emotion regulation and social knowledge in youth at clinical high‐risk for psychosis and outpatients with chronic schizophrenia: Associations with functional outcome and negative symptoms

**DOI:** 10.1111/eip.13287

**Published:** 2022-04-01

**Authors:** William G. Macfie, Michael J. Spilka, Lisa A. Bartolomeo, Cristina M. Gonzalez, Gregory P. Strauss

**Affiliations:** ^1^ Department of Psychology University of Georgia Athens Georgia USA

**Keywords:** attenuated psychosis syndrome, emotion regulation, psychosis, social cognition, ultra high‐risk

## Abstract

**Aim:**

Previous studies indicate that several aspects of social cognition are associated with poor social and vocational outcome in the chronic phase of psychosis. However, it is less clear whether specific aspects of social cognition are impaired in those at clinical high‐risk (CHR) for psychosis and associated with functioning. The current study evaluated two understudied components of social cognition, emotion regulation knowledge and social knowledge, to determine whether CHR and chronic schizophrenia (SZ) samples demonstrated comparable magnitudes of impairment and associations with functioning.

**Methods:**

Two studies were conducted. Study 1 included *n* = 98 outpatients with chronic SZ and *n* = 88 demographically matched healthy controls (CN). Study 2 included 30 CHR and 30 matched CN participants. In both studies, participants completed the emotion management and social management subtests of the Mayer‐Salovey‐Caruso Emotional Intelligence Test to assess emotion regulation knowledge and social knowledge, respectively. A battery of clinical interviews was also administered, including measures of: role and social functioning, positive symptoms, negative symptoms, disorganization and general symptoms.

**Results:**

Individuals with SZ demonstrated lower emotion management and social management scores than CN participants. CHR demonstrated lower scores in social management than CN but did not display deficits in emotion management. In both studies, reduced social knowledge was associated with worse functioning and negative symptoms.

**Conclusions:**

Findings indicate that deficits in social knowledge are transphasic across the SZ spectrum, and are associated with clinical functioning. Social knowledge may be a novel treatment target for psychosocial interventions.

## 1 INTRODUCTION

Psychotic disorders are a leading medical cause of functional disability worldwide (Charlson et al., 2018). Although functional impairments often become more pronounced during the first episode of psychosis (Chang et al., 2016), it is also common to observe declining social and role function during the prodromal (i.e., pre‐illness) period among individuals who meet the criteria for an attenuated psychosis syndrome (APS) (Carrión et al., 2013). Functional impairments therefore reflect a critical treatment target across phases of psychotic illness. Thus, it is of considerable importance to identify mechanisms that underlie social and role dysfunction that may serve as targets for novel interventions.

In the chronic phase of schizophrenia (SZ), deficits have been observed in several domains of social cognition, such as theory of mind, social perception, social knowledge, attributional bias and emotional processing (Green et al., 2008; Green et al., 2015; Savla et al., 2013). Previous studies indicate that several of these domains of social cognition are associated with poor role and/or social functioning (Couture et al., 2006; Fett et al., 2011; Harvey, Strassnig & Silberstein, 2019). Social cognition training programs developed to ameliorate these deficits have been found to be efficacious with some indication of corresponding improvements in functional outcome; however, issues with methodological heterogeneity across studies have also been raised (Combs et al., 2007; Grant et al., 2017; Horan & Green, 2019; Kurtz et al., 2016; Kurtz & Richardson, 2012).

Fewer studies have examined social cognition in those at clinical high‐risk for (CHR) psychosis who meet criteria for a prodromal syndrome. Nonetheless, results of these studies indicate the presence of reduced performance across domains of social cognition in CHR relative to healthy controls (CN), but with generally smaller effect sizes than those found in first‐episode and chronic SZ (Lee et al., 2015; van Donkersgoed et al., 2015; Thompson et al., 2011; but see Green et al., 2012 and Piskulic et al., 2016 for findings of comparable and stable social cognitive difficulties between CHR and first‐episode samples). There is also evidence that difficulties in some domains of social cognition (e.g., theory of mind and facial affect recognition) are associated both with functional outcomes (Barbato et al., 2013; Cotter et al., 2017; Glenthøj et al., 2016) and conversion to psychosis in CHR (Addington et al., 2017; Bora & Pantelis, 2013; Choi & Kwon, 2006; Corcoran et al., 2015; Healey et al., 2013; Kim et al., 2011); however, existing studies are limited and findings have been mixed (Lee et al., 2015; van Donkersgoed et al., 2015). However, the above findings have not translated into the development of extensive social cognition training programs in CHR, and it remains of considerable interest to determine whether there are additional domains of social cognition that are impaired in this population and whether such difficulties are associated with functional outcome.

Two critical domains of social cognition are emotion regulation knowledge and social knowledge. Emotion regulation knowledge is an important aspect of social cognition that reflects the ability to identify contextually appropriate emotion regulation strategies (e.g., savouring positive emotion rather than suppressing it to preserve pleasant mood), whereas social knowledge is defined as the awareness of various roles, goals and rules that typify social situations and interactions (e.g., congratulating a coworker on their promotion rather than ignoring them in order to maintain relationships at work) (Green et al., 2008; Mayer et al., 2002). Prior studies demonstrate deficits in emotion regulation knowledge and social knowledge in SZ as measured by tasks such as the Mayer‐Salovey‐Caruso Emotional Intelligence Test ‘managing emotions’ module (MSCEIT; Mayer et al., 2002) and others (Kee et al., 2009; Savla et al., 2013). Moreover, deficits in emotion regulation and social knowledge have been associated with negative symptom severity and poor functional outcome in SZ (Kee et al., 2009).

Although emotion regulation and social knowledge deficits have consistently been observed in those with SZ, to our knowledge few studies have specifically examined these aspects of social cognition in CHR. For example, in a meta‐analysis of social cognition in CHR (Lee et al., 2015), only 2 of 22 included studies assessed the managing emotions module of the MSCEIT (encompassing emotion regulation and social knowledge), with one study reporting comparable deficits across CHR, first‐episode and chronic SZ groups (Green et al., 2012), while the other reported no differences between CHR and control groups (Thompson et al., 2012). Moreover, these studies reported composite performance scores that did not distinguish between the emotion regulation and social knowledge subtests of this task.

The current manuscript reports findings from a two‐experiment study designed to examine unique deficits in emotion regulation knowledge and social knowledge in outpatients with chronic SZ (study 1) and participants at CHR for psychosis (study 2). It was hypothesized that both SZ and CHR groups would display reduced emotion regulation knowledge and social knowledge compared to control participants, and that these deficits would be associated with worse community‐based role and social functioning. Exploratory analyses were also conducted to examine associations with other clinical domains, including positive, negative, disorganized and general symptoms.

## 2 STUDY 1

### 2.1 Participants

Participants included 98 outpatients meeting DSM‐IV‐TR or DSM‐V criteria for schizophrenia or schizoaffective disorder (SZ) and 88 CN. SZ participants were recruited from the University of Georgia as well as the State University of New York at Binghamton, USA. Diagnosis was made through a combination of psychiatric history and the structured clinical interview for the DSM‐IV (SCID‐IV; First et al., 2002) or the structured clinical interview for DSM‐5 (SCID‐5; First, Williams, Benjamin, et al., 2015). All SZ patients were in the chronic phase of illness and determined to be psychiatrically stable as indicated by no change in medication dose or type within the past 4 weeks.

Healthy control participants (CN) were recruited in the same communities as the patients via online and printed advertisements. CN participants did not meet criteria for any current major psychiatric illnesses (i.e., mood, anxiety, obsessive compulsive disorders, etc.) or SZ‐spectrum personality disorders as determined by SCID‐5 (First, Williams, Benjamin, et al. 2015) and SCID‐5‐PD (First, Williams, Karg, et al., 2015). CN also did not report a family history of psychosis or meet lifetime criteria for a psychotic disorder. Additional exclusion criteria for both groups were: substance dependence within the last 6 months or lifetime history of neurological disorders.

CN and SZ groups did not significantly differ on age, sex, race or parental education; however, SZ had lower personal education than CN (see Table 1).

**TABLE 1:** Participant demographics in study 1 and study 2

	SZ (*n = 98*)	CN (*n = 88*)	Test statistic, *p* value
Age	39.70 (12.49)	39.70 (11.63)	*F* = .00, *p* = .98
Education	13.21 (2.18)	15.36 (2.48)	*F* = 37.20, *p* = .00
Parental education	13.53 (2.58)	14.19 (2.57)	*F* = 2.66, *p* = .11
% Female	50%	58.1%	*χ2* = 1.22, *p* = .27
Race			*χ2* = 12.52, *p* = .05
White	67.3%	59.3%	
Black	21.4%	17.4%	
Biracial	6.1%	4.7%	
Latinx	4.1%	10.5%	
Asian	0%	0%	
Other	0%	2.3%	
BNSS total	17.77 (14.80)	–	
BNSS anhedonia	1.61 (1.56)	–	
BNSS asociality	1.59 (1.52)	–	
BNSS avolition	2.07 (1.73)	–	
BNSS blunted affect	1.42 (1.63)	–	
BNSS alogia	0.63 (1.25)	–	
LOF total	21.56 (7.87)	–	
LOF social	5.92 (2.58)	–	
LOF work	2.90 (3.08)	–	
BPRS positive	2.64 (1.29)	–	
BPRS negative	1.90 (1.08)	–	
BPRS disorganized	2.05 (0.75)	–	
BPRS total	43.12 (10.05)	–	

	CHR (*n = 30*)	CN (*n = 30*)	Test statistic, *p* value
Age	21.93 (3.34)	21.40 (2.40)	*F* = .50, *p* = .48
Education	13.90 (1.73)	14.58 (1.49)	*F* = 2.70, *p* = .11
Parental education	15.53 (3.07)	15.45 (1.86)	*F* = .02, *p* = .90
% Female	73.3%	70%	*χ2* = .08, *p* = .77
Race			*χ2* = 5.09, *p* = .41
White	76.7%	70%	
Black	10%	6.7%	
Biracial	3.3%	3.3%	
Latinx	3.3%	6.7%	
Asian	3.3%	16.7%	
Other	3.3%	0%	
NSI‐PR avolition	1.94 (1.11)	–	
NSI‐PR asociality	1.61 (0.85)	–	
NSI‐PR anhedonia	1.32 (1.03)	–	
NSI‐PR blunted affect	0.76 (1.19)	–	
NSI‐PR alogia	0.37 (1.07)		
GFS: Social	7.73 (1.44)	–	
GFS: Role	7.47 (1.46)	–	
SIPS positive	2.33 (0.64)	–	
SIPS negative	1.33 (0.91)	–	
SIPS disorganized	1.32 (0.66)	–	
SIPS general	2.23 (1.10)	–	

*Note*: BNSS, BPRS, NSI‐PR, and SIPS subscores reflect the mean individual item score within each symptom domain.

Abbreviations: BNSS, brief negative symptom scale; BPRS, brief psychiatric rating scale; CHR, clinical high‐risk group; CN, control group; GFS, global functioning scale; LOF, level of function scale; NSI‐PR, negative symptom inventory psychosis‐risk scale; SIPS, structured interview for psychosis‐risk syndromes; SZ, schizophrenia group.

### 2.2 Procedure

Study procedures were approved by the local institutional review board. All participants provided written informed consent and received monetary compensation for participation in the study. All clinical interviews were conducted by a licensed clinical psychologist or raters trained to reliability standards on each measure (alpha ≥ 0.80). SZ participants were rated on the brief negative symptom scale, (BNSS: Kirkpatrick et al., 2010), brief psychiatric rating scale (BPRS; Overall & Gorham, 1962) and level of function scale (LOF; Hawk et al., 1975). Participants also completed the MATRICS consensus cognitive battery (MCCB; Nuechterlein et al., 2008) which includes subtests from the MSCEIT (Mayer et al., 2002).

Two MCCB MSCEIT subtests were used to assess emotion regulation knowledge and social knowledge, respectively: emotion management and social management. In the emotion management subtest, the examiner reads aloud a series of brief vignettes about positively or negatively valenced situations, and the participant is asked to rate the effectiveness of possible actions for regulating one's own emotional state. In the social management subtest, the vignettes are social scenarios and the participant is asked to rate how different actions would impact relationships with others. For both subtests, each vignette includes several multiple‐choice response options, and the participant is asked rate each option on a 5‐point scale ranging from 1 (very ineffective) to 5 (very effective). Standard scores were calculated for each subtest via the standard MCCB scoring software using the age and gender norms, where the average score is 100 and the SD is 15. The MSCEIT managing emotions overall score was also calculated for the subtest scores and reported in Table 2. Higher scores indicate better emotion or social management.

**TABLE 2:** Emotion regulation and social knowledge in schizophrenia and clinical high‐risk groups compared to controls

	SZ (*n =* 98)	CN (*n =* 86)	Test statistic, *p* value
Emotion management	93.50 (10.90)	97.54 (10.10)	*F* = 6.72, *p* = .01
Social management	91.94 (10.25)	96.38 (9.18)	*F* = 9.45, *p* = .002
Overall score	91.96 (10.62)	97.21 (9.76)	*F* = 12.06, *p* = .001

	CHR (*n =* 30)	CN (*n =* 30)	Tet statistic, *p* value
Emotion management	94.65 (8.36)	97.79 (7.03)	*F* = 2.48, *p* = .12
Social management	92.14 (8.87)	99.56 (5.41)	*F* = 15.31, *p* = .00
Overall score	92.98 (8.71)	99.29 (4.79)	*F* = 12.12, *p* = .001

Abbreviations: CHR, clinical high‐risk group; CN, control group; SZ, schizophrenia group.

### 2.3 Data analysis

Group differences in social cognition were analysed using two separate one‐way analysis of variance (ANOVAs) using the emotion management and social management MSCEIT subtests. Bivariate correlations were used to determine associations between MSCEIT scores and measures of social and work functioning. Exploratory bivariate correlations were also conducted between the 2 MSCEIT subscales and measures of positive symptoms, negative symptoms, disorganization and general symptoms.

## 3 RESULTS

One‐way ANOVAs indicated that SZ scored significantly lower on the emotion management subscale and the social management subscale relative to CN (see Table 2).

Lower emotion management scores were significantly associated with more severe global functional impairment, asociality, positive symptoms and disorganized symptoms. Lower social management scores were significantly associated with more severe negative symptoms, including anhedonia, asociality, avolition, blunted affect and alogia. Associations with other clinical outcomes were nonsignificant (see Table 3).

**TABLE 3:** Correlations between social cognition and clinical outcomes in study 1 (schizophrenia)

	Emotion management	Social management
BNSS total	−0.17	−0.35**
BNSS anhedonia	−0.13	−0.24*
BNSS asociality	−0.27**	−0.39**
BNSS avolition	−0.18	−0.26*
BNSS blunted affect	−0.06	−0.24*
BNSS alogia	−0.17	−0.28**
LOF total	0.24*	0.36**
LOF social	0.18	0.29**
LOF work	0.17	0.35**
BPRS positive symptoms	−0.26*	−0.37**
BPRS negative symptoms	−0.07	−0.19
BPRS disorganized symptoms	−0.31**	−0.17
BPRS total	−0.21*	−0.28**

*Note*: ** Denotes correlation is significant at the *p <* .01 level (2‐tailed). * Denotes correlation is significant at the *p* < .05 level (2‐tailed).

Abbreviations: BNSS, brief negative symptom scale; BPRS, brief psychiatric rating scale; LOF, level of function scale.

## 4 STUDY 2

### 4.1 Participants

Participants included 30 individuals at CHR for psychosis and 30 CN (see Table 1). CHR participants were recruited from the Georgia Psychiatric Risk Evaluation Program (G‐PREP), consisting of clinician‐referred individuals with emerging psychotic experiences who undergo diagnostic and symptom monitoring assessments. Additional CHR participant recruitment occurred through local advertisements (print and online), in‐person presentations to community mental health centers, and meetings/presentations with the local school system. CHR status was determined by the structured interview for prodromal syndromes (SIPS; Miller et al., 2003). CHR participants were included in the study if they met SIPS criteria for progression or persistence on the attenuated positive symptom psychosis risk syndrome (*n* = 13 persistence, 17 progression).

CN participant recruitment occurred through posted flyers and electronic advertisements in the local community. Exclusion criteria for CN participants were identical to Study 1. CN and CHR did not significantly differ on age, sex, ethnicity and personal or parental education (see Table 1).

### 4.2 Procedure

Participants provided written informed consent and received monetary compensation for their participation. Study procedures were approved by the University of Georgia Institutional Review Board. Participants completed a structured clinical interview to rate the SCID‐IV (First, Williams, Benjamin, et al., 2015), SIPS (Miller et al., 2003), negative symptom inventory psychosis‐risk scale (NSI‐PR; Strauss et al., 2020), BNSS (Kirkpatrick et al., 2010; Strauss & Chapman, 2018) global functioning scale: social (GFS:S, Cornblatt et al., 2007) and global functioning scale: role (GFS:R; Cornblatt et al., 2007). Participants completed the MATRICS consensus cognitive battery (MCCB; Nuechterlein et al., 2008), which includes the MSCEIT emotion management and social management subtests, as in study 1.

### 4.3 Data analysis

The analytic plan was identical to study 1, with alternate measures used to assess clinical outcome in correlations.

## 5 RESULTS

One‐way ANOVAs indicated that CHR did not significantly differ from CN on the emotion management subscale, but did score significantly lower on the social management subscale (see Table 2).

Correlations between Emotion Management scores and clinical and functional outcomes were nonsignificant. Lower scores on the Social Management subscale were associated with more severe asociality, anhedonia and greater impairment in role functioning (see Table 4).

**TABLE 4:** Correlations between social cognition and clinical outcomes in study 2 (clinical high‐risk)

	Emotion management	Social management
NSIPR avolition	−0.15	−0.39*
NSIPR asociality	0.23	−0.21
NSIPR anhedonia	−0.05	−0.42*
NSIPR blunted affect	0.10	−0.10
NSIPR alogia	0.04	−0.25
GFS social	−0.34	0.07
GFS role	0.20	0.54**
SIPS positive	0.12	−0.06
SIPS negative	0.19	−0.08
SIPS disorganized	0.22	0.17
SIPS general	0.23	−0.18

*Note*: * Denotes correlation is significant at the *p* < .05 level (2‐tailed). ** Denotes correlation is significant at the *p* < .01 level (2‐tailed).

Abbreviations: GFS: global functioning scale; NSI‐PR, negative symptom inventory psychosis‐risk scale; SIPS, structured interview for psychosis‐risk syndromes.

## 6 DISCUSSION

The current study examined whether outpatients with chronic SZ and participants at CHR for psychosis displayed deficits in emotion regulation knowledge and social knowledge on the MSCEIT in comparison to CN, and whether such difficulties were associated with social and role functioning and clinical symptoms.

Consistent with past studies, individuals with SZ displayed deficits on the emotion and social management subtests relative to CN (Kee et al., 2009). These deficits were associated with reduced role and social functioning and more severe asociality, positive symptoms and disorganization, consistent with prior studies indicating similar associations with clinical ratings and functional outcome (Fett et al., 2011; Horan et al., 2012; Kee et al., 2009). Such findings highlight the importance of targeting emotion regulation and social knowledge in social cognitive training programs, which have shown efficacy in several studies (Kurtz et al., 2016; Kurtz & Richardson, 2012; Roberts & Penn, 2009).

Importantly, we also extended prior findings by characterizing emotion regulation knowledge and social knowledge abilities in CHR. Compared to CN, CHR participants displayed deficits on the social management subtest, but not the emotional management subtest. Furthermore, reduced social management performance, but not emotion management performance, was associated with greater severity of negative symptoms and with reduced role functioning. These findings point to differentially affected aspects of social cognition in the CHR population, underscoring the importance of fine‐grained distinctions between multiple components of social cognition to improve our understanding of the CHR cognitive profile and clinical correlates.

Social knowledge is considered to be a building block for successful social interactions, in that one must possess knowledge of the rules governing social situations to identify and respond appropriately to social cues (Green et al., 2008). However, interestingly, social knowledge deficits were associated with reduced role (i.e., educational/occupational) but not reduced social functioning in the CHR sample. Social knowledge may be particularly relevant to role functioning due to the more formalized rules, goals and roles in educational and occupational contexts. Additionally, in both CHR and SZ groups, social knowledge deficits were associated with greater negative symptoms. The significant association between social management scores and avolition and anhedonia may indicate that negative symptoms impede CHR youth from applying social management strategies, such that they may lack the motivation to do so or do not find it rewarding. However, deficits in motivation and pleasure may only impact functioning within specific contexts, as evidenced by the significant association between social management scores and role but not social functioning. One possibility is that CHR youth may have developed compensatory mechanisms to effectively function outside of role contexts (e.g., with friends, family and romantic partners). Further, social relationships are more informal and flexible and may be less impacted by negative symptoms. Alternatively, the participants' social networks may consist of other people with similar or higher levels of social management knowledge that compensate for their personal difficulties. Identifying compensatory factors acting on the relationship between social management deficits and social functioning in CHR is thus an important direction of future research. Existing research suggests both shared and distinct mechanisms for social cognitive deficits and negative symptoms in SZ (Mancuso et al., 2011; Pelletier‐Baldelli & Holt, 2020). Further, future studies into the emergence of these difficulties in CHR and other ‘at risk’ samples may help clarify etiological mechanisms for these difficulties in individuals with psychosis and identify novel intervention targets.

The findings of preserved emotion regulation knowledge in CHR are in contrast to several studies documenting dysfunction in emotional awareness and emotion regulation strategy use assessed via self‐report in CHR (e.g., Chapman et al., 2020; Kimhy et al., 2016). It may be that although CHR individuals possess adequate knowledge of appropriate emotion regulation strategies for a given situation, they have difficulty successfully applying or benefiting from these strategies in the moment. Indeed, there is neurophysiological evidence of inefficient emotion regulation in response to unpleasant emotional images both in SZ (Bartolomeo et al., 2020; Strauss et al., 2013; Strauss et al., 2015) and CHR (Kim et al., 2021). Future studies employing ecological momentary assessment approaches may help address these gaps in understanding by examining emotion regulation knowledge and effectiveness in real‐world contexts (Raugh & Strauss, 2021; Visser et al., 2018).

The current findings should be interpreted in light of certain limitations. First, the study design was cross‐sectional. We did not have longitudinal data available on the CHR sample to determine whether emotion regulation and social knowledge predict conversion to illness. Second, although the SZ sample and comparison group were adequately powered, the CHR sample and their CN group were modest in size, resulting in lower power to detect the potential presence of a smaller effect for emotion regulation knowledge in the CHR group. Third, the current study focused specifically on emotion regulation knowledge and social knowledge, and we did not assess additional domains of social cognition of potential relevance to social cognitive remediation efforts in CHR (e.g., theory of mind). Fourth, although emotion regulation knowledge and social knowledge performance were assessed separately, these processes cannot be fully separated and likely partially overlap with other social cognitive domains.

Despite these limitations, the current findings provide evidence for transphasic deficits in social knowledge in SZ. Deficits in social knowledge were also associated with a number of clinical variables across phases of illness, suggesting that they may reflect novel targets for psychosocial or social cognition training interventions.

## CONFLICT OF INTEREST

Gregory P. Strauss is one of the original developers of the brief negative symptom scale (BNSS) and receives royalties and consultation fees from Medavante‐ProPhase LLC in connection with commercial use of the BNSS and other professional activities; these fees are donated to the Brain and Behaviour Research Foundation. Gregory P. Strauss has received honoraria and travel support from ProPhaseLLC for training pharmaceutical company raters on the BNSS. Gregory P. Strauss has consulted for minerva neurosciences, acadia and lundbeck. All other authors have no relevant disclosures.

## Data Availability

Data is available upon request from the corresponding author.
